# Molecular Basis for Vitamin A Uptake and Storage in Vertebrates

**DOI:** 10.3390/nu8110676

**Published:** 2016-10-26

**Authors:** Sylwia Chelstowska, Made Airanthi K. Widjaja-Adhi, Josie A. Silvaroli, Marcin Golczak

**Affiliations:** 1 Department of Pharmacology, School of Medicine, Case Western Reserve University, Cleveland, OH 44106, USA; sxc968@case.edu (S.C.); nkw5@case.edu (M.A.K.W.-A.); jxs1329@case.edu (J.A.S.); 2 Laboratory of Hematology and Flow Cytometry, Department of Hematology, Military Institute of Medicine, PL 04-141 Warsaw, Poland; 3 Cleveland Center for Membrane and Structural Biology, School of Medicine, Case Western Reserve University, Cleveland, OH 44106, USA

**Keywords:** vitamin A, retinol, lecithin:retinol acyltransferase (LRAT), lipid metabolism, visual cycle

## Abstract

The ability to store and distribute vitamin A inside the body is the main evolutionary adaptation that allows vertebrates to maintain retinoid functions during nutritional deficiencies and to acquire new metabolic pathways enabling light-independent production of 11-*cis* retinoids. These processes greatly depend on enzymes that esterify vitamin A as well as associated retinoid binding proteins. Although the significance of retinyl esters for vitamin A homeostasis is well established, until recently, the molecular basis for the retinol esterification enzymatic activity was unknown. In this review, we will look at retinoid absorption through the prism of current biochemical and structural studies on vitamin A esterifying enzymes. We describe molecular adaptations that enable retinoid storage and delineate mechanisms in which mutations found in selective proteins might influence vitamin A homeostasis in affected patients.

## 1. Introduction

Vitamin A is an essential fat-soluble nutrient with a pivotal role in various metabolic and physiological processes within the body [1,2]. Among others, it is essential for vision, embryonic development, normal growth and development of infants, and immunity [3,4,5,6,7]. With the exception of vision, the physiological actions of vitamin A are mediated by its bioactive metabolite, retinoic acid [8,9,10]. Through binding to retinoic acid receptors (RARs) and retinoid X receptors (RXRs), this ligand regulates target gene transcription [11,12]. Retinoic acid can enhance or decrease expression of more than 500 genes depending on the target cell type and the physiological state of the organism [12,13].

The complex and multidimensional physiological function of vitamin A and its metabolites requires the maintaining of retinoids at defined and well-controlled levels. The importance of proper retinoid homeostasis is underscored by the fact that both an excess and deficiency of vitamin A lead to detrimental consequences particularly during development. Excess of vitamin A may result in teratogenic effects, lethargy, bone fractures, cortical thickening, and several immunopathology conditions such as respiratory tract infections and autoimmune disease [14,15,16,17]. Retinoid deficiencies are the leading cause of preventable blindness, growth stunting, increased morbidity, delayed cognitive functions, and thus a significant contributor to mortality and morbidity for children and pregnant women that claim the lives of 670,000 children under 5 annually [18,19,20]. Because animals cannot synthesize retinoids de novo, they solely depend on a dietary source of vitamin A obtained as either pre-formed retinoids or pro-vitamin A carotenoids, of which the most abundant representatives are retinyl esters (REs) or β-carotene, respectively [21]. To alleviate dependence on the fluctuating external supply of these essential nutrients, vertebrates evolved complex strategies to store vitamin A in the form of REs. These stores can be mobilized depending on the retinoid demand of the organism. This evolutionary adaptation provided a key pro-survival advantage as well as enabled development of new metabolic pathways such as enzymatic regeneration of the visual chromophore (11-*cis*-retinal), critical for advance visual performance of a vertebrate eye.

In this review, we summarize the current knowledge regarding absorption, storage, and mobilization of vitamin A with special emphasis on new insights for molecular mechanisms of vitamin A esterification gained through advances of structural biology.

## 2. Overview of the Retinoid Absorption, Storage, and Transport to the Peripheral Tissues

**Figure 1:**
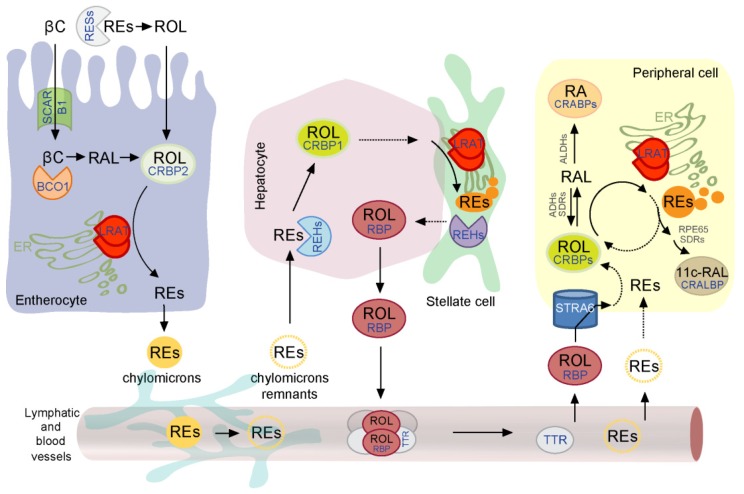
Diagram of the current model of vitamin A absorption, transport, and storage. Retinoid metabolism can be classified into three major processes—intestinal uptake, hepatic storage, and tissue-specific metabolism that are interconnected via lymphatic and blood vitamin A transport. Although each of these steps is characterized by a set of specialized proteins, lecithin:retinol acyltransferase (LRAT) plays a pivotal role in each of them. A detailed description of the pathways can be found in Section 2 of this review. The abbreviations used are the following: 11c-RAL, 11-*cis*-retinal; ADHs, alcohol dehydrogenases; ALDHs, aldehyde dehydrogenases; βC, β,β-carotene; BCO1, β,β-carotene-15,15-dioxygenase; CRABPs, cellular retinoic acid-binding proteins; CRALBP, cellular retinaldehyde-binding protein; CRBP1, cellular retinol-binding protein 1; CRBP2, cellular retinol-binding protein 2; ER, endoplasmic reticulum; LRAT, lecithin:retinol acyltransferase; RA, all-*trans*-retinoic acid; RAL, all-*trans*-retinaldehyde; REHs, retinyl ester hydrolases; REs, retinyl esters; RESs, retinyl esterases; RPE65, retinoid isomerase; ROL, all-*trans*-retinol; RBP, serum retinol-binding protein; SCARB1, scavenger receptor class B, type I; SDRs, short-chain dehydrogenases/reductases; STRA6, stimulated by retinoic acid 6; TTR, transthyretin.

Unlike other fat-soluble vitamins, for which relatively small amounts (several pmol/g) can be found predominantly in the body’s adipose tissues, vitamin A can be stored in high quantities ranging between 10–1000 nmol/g mainly in liver but also in kidneys and lungs [22]. This pool of retinoids exists mostly in the form of chemically inert REs and can be mobilized depending on the body’s demand for vitamin A [23,24,25]. The ability to retain vitamin A within certain tissues would not be possible without a specialized metabolic pathway that involves retinoid-binding proteins, enzymes, and receptors. Understanding the molecular principles, which underlie uptake, storage, and mobilization of vitamin A requires identification of the key protein components involved in these processes (Figure 1).

### 2.1. Role of Vitamin A Esterification in the Intestinal Uptake

The proximal part of the small intestine is the primary site where the dietary retinoids (predominantly all-*trans*-retinol and its fatty acids esters) and pro-retinoid carotenoids are absorbed [26]. Retinol is taken up directly by the enterocytes via passive non-ionic diffusion through the Brush border membrane, whereas REs need to be hydrolyzed in the intestinal lumen or at the Brush membrane prior to the absorption of the resulting retinol [27,28]. The ester bond hydrolysis is catalyzed predominantly by a synergistic action of pancreatic enzymes triglyceride lipase and lipase-related protein 2, and intestinal phospholipase B [28,29]. Unlike retinol, carotenoids uptake is tightly controlled. It depends on scavenger receptor class B, type I (SCARB1), which is a receptor for high-density lipoproteins that also facilitates the intestinal absorption of other lipophilic compounds including tocopherols [30,31]. Interestingly, the expression level of SCARB1 in enterocytes is regulated by the homeodomain transcriptional factor ISX (intestinal specific homeobox) in a retinoic acid-dependent manner [32]. ISX is present in the epithelia of the intestine and its expression is readily detectable as early as mouse embryonic day 14 [33]. Besides SCARB1, ISX also controls the expression levels of β,β-carotene-15,15-dioxygenase (BCO1), a key enzyme in carotenoid metabolites that catalyzes symmetrical cleavage of the polyene chain of β-carotene yielding two molecules of retinal [34]. Thus, efficiency of carotenoids absorption is controlled by a negative feedback, in which ISX suppresses the intestinal expression of SCARB1 and the vitamin A forming enzyme BCO1 via retinoic acid signaling directly linked with the vitamin A demand of the organism [33].

The effective passive diffusion of retinol or the protein-mediated transport of carotenoids depends on the concentration gradient across the enterocyte membrane. To create and maintain such a gradient and to generate a sufficient driving force for absorption, retinol needs to be continuously removed from the system. Thermodynamically, this can be achieved in two ways: (a) by phase separation that involves binding of retinol to specific soluble carrier proteins; or (b) enzymatic conversion of retinol into its metabolites. The enterocytes utilize both strategies. To facilitate the solubilization and cytosolic transport of water insoluble vitamin A, retinol derived from carotenoids or REs binds to specific cellular retinoid-binding proteins. A subtype of such a carrier protein, cellular retinol-binding protein 2 (CRBP2) is highly expressed in enterocytes representing up to 1% of total cytosolic proteins [35,36]. The main function of CRBP2 is to transport vitamin A across the cytoplasm to the endoplasmic reticulum, where retinol becomes esterified by the action of two non-related proteins: lecithin:retinol acyltransferase (LRAT) and diacylglycerol *O*-acyltransferase 1 (DGAT1) [37]. Although the final products of their enzymatic activity are REs, these two enzymes differ fundamentally in terms of enzymatic strategies. LRAT catalyzes the transesterification of retinol utilizing a fatty acyl moiety present in the sn-1 position of phosphatidylcholine [38,39,40], whereas DGAT1 depends on a pre-activated form of fatty acid carried by acyl-CoA [41]. Additionally, in terms of substrate specificity, LRAT activity is limited to retinol and its derivatives such as endogenous apo-carotenoids or xenobiotic retinylamine that share a common structural motif [42,43]. DGAT1, however, exhibits much broader substrate specificity and transfers acyl moieties onto alternative substrates, including 1,2-diacylglycerol, contributing to the formation of triacylglycerols [44]. This substrate promiscuity is reflected in the biological functions of these two enzymes. Under physiological conditions, LRAT activity accounts for approximately 90% of REs formed in enterocytes, the remaining 10% is contributed by DGAT1 [25]. Therefore, esterification of retinol by LRAT is the main driving force behind vitamin A absorption in the small intestine. 

### 2.2. LRAT Activity in Hepatic Retinoid Metabolism

Esterified dietary vitamin A is integrated with other dietary lipids into nascent chylomicrons and secreted by enterocytes to the lymphatic system. Approximately 70% of chylomicrons are uptaken by the liver, the main organ involved in storage of retinoids and regulation of serum vitamin A levels [45,46]. The remaining chylomicrons are cleared out by other peripheral tissues. Incorporation of chylomicron remnants by hepatocytes is a receptor-mediated process involving the low density lipoprotein receptor [47]. Internalized REs undergo rapid hydrolysis to liberate retinol. Numerous hepatic enzymes exhibit the ability to cleave REs, among them carboxyl ester lipases, carboxylesterases, and hepatic lipases [48,49,50,51,52]. However, it is unclear at this moment whether they act synergistically or whether certain classes of these enzymes play a dominant role in this process. 

From the hepatocytes, vitamin A is transported to a specific subset of liver cells named hepatic stellate cells (HSCs) in a manner that is not fully understood [53,54]. HSCs are much smaller and less abundant than hepatocytes and represent only about 15% of total liver cells [55]. However, they play a central role in the uptake, storage, and mobilization of hepatic vitamin A. The most eminent feature of HSCs is the presence of lipid droplets that contain 90%–95% of hepatic retinoids in addition to other non-retinoid lipids [56,57,58]. LRAT is solely responsible for vitamin A esterification in HSCs and formation of the lipid droplets [25,59,60]. Studies on homozygous *Lrat* knock-out (*Lrat^−/−^*) mice revealed undetectable levels of RE in the liver extracts ruling out plausible contributions of DGAT1 or other acyl-CoA-dependent transferases in hepatic retinol esterification. The amount of REs stored in HSCs strongly correlates with the dietary vitamin A and carotenoids supply underscoring the regulatory role of these cells in maintaining retinoid homeostasis [23].

### 2.3. Mobilization of Retinol from the Liver Storage and LRAT-Driven Uptake by the Extrahepatic Tissue

The signaling cascade that leads to mobilization of hepatic storage of REs in response to the retinoid status of the peripheral tissue is not currently understood. However, it is clear that retinoid recruitment requires hydrolysis of REs back to retinol. Several candidate lipases expressed in HSCs exhibit the ability to cleave the ester bond of REs, among them hepatic carboxylesterases 2, 4, and 10 [61,62,63]. Following the release, retinol is transported back to hepatocytes, where it binds to a specific 21-kDa retinol-binding protein, RBP [64,65,66]. Retinol-bound (holo-) RBP is subsequently secreted into circulation. The significance of RBP-mediated vitamin A transport is underscored by the fact that in a fasting state more than 95% of total retinol present in the blood serum is found in RBP-bound form. To prevent infiltration through the kidneys, holo- but not apo-RBP associates with transthyretin (TTR) [67]. This plasma protein forms a 55 kDa homotetramer with a dimer of dimers quaternary structure and binds up two molecules of holo-RBP forming a relatively large 75 kDa complex [68]. 

**Figure 2:**
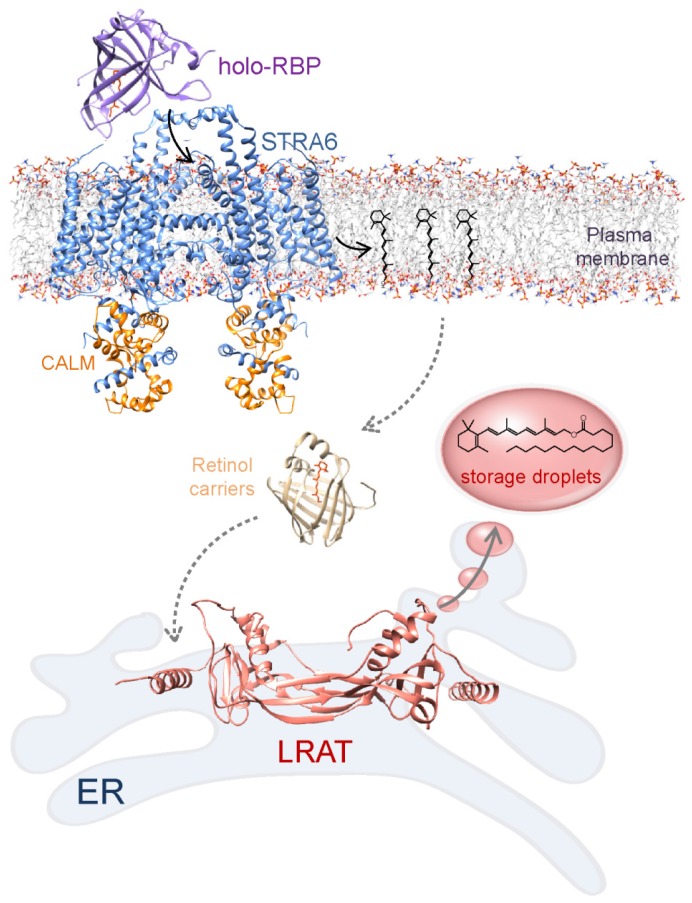
Schematic representation of STRA6-dependent vitamin A cellular uptake. Transport of all-*trans*-retinol carried by serum retinol-binding protein (RBP) across the plasma membrane is mediated by RBP receptor, STRA6. On the cytoplasmic side, vitamin A binds to retinol binding proteins. They might facilitate transport of retinol across the cytoplasm to the endoplasmic reticulum (ER), where it is esterified by action of lecithin:retinol acyltransferase (LRAT). LRAT acts independently in storing vitamin A and cellular retinol binding proteins are not absolutely essential for its activity. Because the factors that contribute to the transport of vitamin A across the cytoplasm are not fully understood this process is illustrated by dashed arrows. Structures of RBP and retinol binding protein (here cellular retinol-binding protein 1, CRBP1) correspond to PDB accession numbers 1RBP [78] and 5H8T [79], respectively. STRA6 refers to the cryo-electron microscopy structure of zebrafish protein in complex with calmodulin (EM Data Bank #8315) [75]. LRAT model was built base on the structure of HRASLS3/LRAT chimera (PDB accession numbers 4Q95) [80].

Delivery of RBP bound vitamin A to specific tissues is mediated by a RBP receptor called STRA6 (stimulated by retinoic acid 6) (Figure 2) [69,70,71]. This integral membrane protein is expressed in numerous tissues known to be involved or dependent on retinoid metabolism with the exception of liver and adipocytes [69,72]. The extracellular portion of STRA6 recruits holo-RBP allowing retinol to pass through the receptor to the plasma membrane [73,74,75]. This process requires neither energy from hydrolysis of ATP nor synergistic co-transport of other molecules across the lipid membrane [76]. It is also not dependent on endocytosis of the RBP-STRA6 complex. Instead, efficient absorbance of retinol via STRA6 involves functional coupling to two intracellular proteins: cellular retinol-binding protein 1 (CRBP1) and LRAT [70,76]. Although STRA6 constitutes a functional unit on its own and exhibits activity independent from the coupling proteins, apo-CRBP1 stimulates STRA6’s activity by increasing vitamin A binding capacity in the cytoplasm [76]. Nevertheless, binding of retinol by apo-CRBP1 does not provide a sufficient driving force for enabling efficient retinoid uptake. Numerous lines of evidence including experiments performed in isolated systems, cell lines as well as in the mouse models indicate that esterification of retinol by LRAT within the cell, and subsequent sequestration of resulting retinyl esters into lipid droplets are responsible for creating a chemical gradient that allows for the uptake [69,70,77]. Remarkably, the recently published 3D structure of STRA6 indicates that retinol entering the cell does not interact with CRBP1 but rather is released by the receptor directly into the plasma membrane [75]. If correct, this model raises an intriguing question regarding factors that facilitate transport of vitamin A between the plasma membrane and the endoplasmic reticulum where LRAT catalyzes the formation of retinyl esters.

## 3. Role of REs in the Ocular Metabolism of Retinoids

Because of their chemical stability and hydrophobicity, REs serve as a transport and storage form of vitamin A and therefore play an essential role in maintenance of retinoid homeostasis. However, they can also be involved more directly in retinoid metabolism by becoming precursors for bioactive forms of vitamin A. The best illustration of this fact is retinyl palmitate’s role in production of the visual chromophore. 

**Figure 3:**
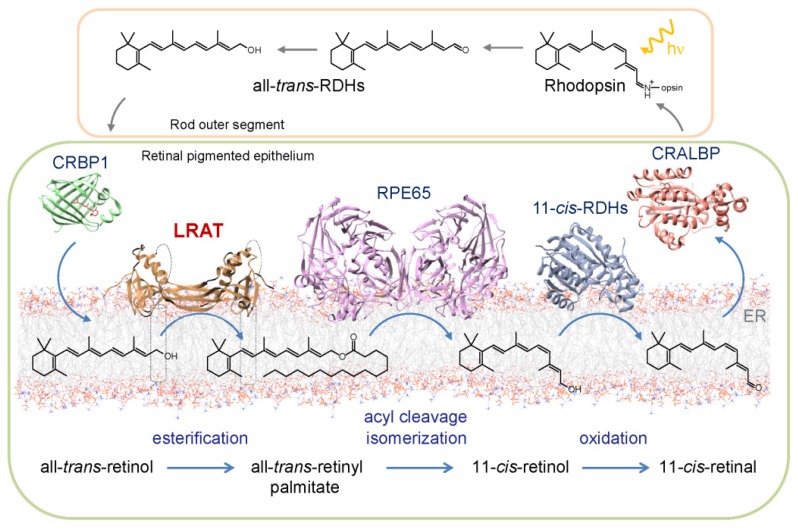
The enzymatic production of the visual chromophore via canonical retinoid (visual) cycle. 11-*cis*-Retinal regeneration is initiated in the photoreceptor by photoisomerization of the chromophore bound to rhodopsin, its subsequent liberation and reduction to all-*trans*-retinol. In RPE cells, all-*trans*-retinol is esterified by LRAT prior to isomerization that is accompanied the acyl cleavage of the ester bond. Resulting 11-*cis*-retinol is then oxidized to the corresponding aldehyde. These metabolic transformations occur in the smooth ER, where key enzymes of the visual cycle are located. The retinoid cycle is completed by transport of 11-*cis*-retinal back to the photoreceptors, where it conjugates with opsin and thus regenerates visual pigment. Structures of cellular retinol-binding protein 1 (CRBP1), retinoid isomerase (RPE65) and cellular retinaldehyde-binding protein (CRALBP) correspond to PDB accession numbers 5H8T [79], 3KVC [96], and 4CIZ [97], respectively. Generic model of 11-*cis*-retinol dehydrogenases (11-*cis*-RDHs) was built based on the structure of dehydrogenase from *Drosophila melanogaster* PDB accession number 5ILG.

The fundamental step in seeing is light-induced 11-*cis* to all-*trans* geometric isomerization of a visual chromophore [81]. In contrast to invertebrates, in which isomerized visual chromophore does not leave the binding pocket and can be re-isomerized back to 11-*cis* configuration in situ [82,83], vertebrates evolved a relatively complex sequence of enzymatic reactions to facilitate thermodynamically unfavorable isomerization of retinoid independently of light [3,84]. This metabolic pathway is called the visual or retinoid cycle (Figure 3). By binding to a protein scaffold (opsin), 11*-cis*-retinal forms a functional visual pigment, rhodopsin [85,86]. This G-protein coupled receptor is capable of capturing and converting a photon into a cascade of chemical signaling events that eventually result in visual perception. Upon release from the activated rhodopsin, all-*trans*-retinal is readily reduced in a NADPH-dependent manner by all-*trans*-retinol dehydrogenases (RDH) present in photoreceptors, particularly RDH8 and RDH12 [87,88,89]. Resulting all-*trans*-retinol is transported to neighboring retinal pigmented epithelium (RPE) cells where it is subsequently esterified by LRAT. Similarly to enterocytes and HSCs, formation of REs in RPE facilitates transport and storage of retinoids. This has two important implications; (a) efficient elimination of excess retinoids from photoreceptors that in turn alleviates the cytotoxic effect of retinal or its condensation products and (b) formation of a readily available ocular pool of vitamin A stored in lipid droplets called retinosomes in the RPE used to regenerate visual pigment during intense light exposure. It remains to be clarified whether REs present in retinosomes serves as the direct substrate for the retinoid isomerase (RPE65 or Retinal pigment epithelium-specific 65 kDa protein) or if the mobilization of REs from lipid droplets requires hydrolysis, transport, and subsequent re-esterification of retinol in the endoplasmic reticulum. In both scenarios, LRAT activity creates the substrate for RPE65 [90]. The enzymatic isomerization reaction combines Fe^2+^-mediated acyl cleavage of the ester bond in retinyl pamitate with isomerization of the retinoid polyene chain at the 11–12 bond yielding free fatty acid and 11-*cis*-retinol as the final products [91]. As evident by biochemical and structural studies, REs cleavage at the C15-O bond and subsequent formation of retinylic cation are critical for lowering ~36 kcal/mol activation energy barrier of double bond isomerization [92]. The visual cycle is completed when newly formed 11-*cis*-retinol is oxidized to the corresponding aldehyde predominantly by RDH5, 11, and 10 and transported back to the photoreceptor cell where it binds to opsin and regenerates functional visual pigment [93,94,95]. 

## 4. Insight into Molecular Adaptations that Enable LRAT Activity in the Vertebrates 

Evolution’s choice of vitamin A as a precursor for the vital signaling molecule (retinoic acid) and the essential visual pigment chromophore (11-*cis*-retinal) triggered selective pressure to advance an efficient system for retinoid storage and transport. Although certain retinoid metabolites characteristic for the metabolic pathways involved in maintaining retinoid homeostasis can be found in early metazoans, the key genes that encode LRAT, RBP, and STRA6 are absent in invertebrate genes [98,99]. The reported presence of REs in tissues of sponges and two gastropods species can instead be attributed to the activity of DGAT1 orthologues [100,101,102]. Moreover, the absence of the capability to recruit REs in several other invertebrate lineages strongly suggests lack of a coherent mechanism for vitamin A storage [103]. Thus, the combined abilities to accumulate, sustainably release, and transport vitamin A to a specific tissue appear to be vertebrates’ sophistication [104,105]. This conclusion is supported by comparative genetic studies on the invertebrate chordate amphioxus (*Branchiostoma floridae*), a representative organism for the most basal subphylum of vertebrates. These analyses failed to identify a vertebrate-like genetic component enabling retinoid storage, transport, and delivery [98]. In fact, the first functional LRAT was cloned from sea lamprey (*Petromyzon marinus*), a jawless vertebrate [104]. Thus, it is tempting to speculate that the ability to store vitamin A inside the cell occurred following evolutionary divergence of the more primitive chordates in the last common ancestor of the jawless and jawed craniates [104,106]. Interestingly, it is not a mere coincidence that the appearance of LRAT concurs with expression of ancestral RPE65. Sea lamprey is the most primitive organism with a fully structured camera like eye containing a lens, iris, and ocular muscles. Thus, these two enzymes originated collaterally to facilitate higher visual performance in dim light and upon sudden changes in light intensity, by enabling enzymatic production of 11-*cis*-retinoids. The above findings raise a fundamental question of what are the molecular adaptations of proteins and enzymes that determine how vertebrates acquire the ability to absorb and store vitamin A. In other words, how does nature engineer novel activities by modifying existing processes to cope better with the environmental challenges? 

Although phylogenetic analyses provide important insight into the evolutionary origin of proteins, understanding changes that lead to enquiring new functions on the molecular level requires detailed structural studies. The recent crystal structure of the chimeric protein of LRAT and HRAS-like tumor suppressor 3 (HRASLS3), a homologous LRAT-like protein family member, has provided a novel insight into the molecular adaptations that permits diversification of the enzymatic activity and thus gain the ability to process vitamin A for vertebrates [80]. 

### 4.1. Evolutional Diversity of N1pC/P60 Protein Family 

Despite being evolutionary ‘young’, LRAT belongs to an ancient and diverse group of proteins called N1pC/P60 [107]. These ubiquitous enzymes, present in all kingdoms of life, utilize a papain-like fold to adapt diverse enzymatic functions. Several related, but distinct, catalytic activities have arisen in the N1pC/P60 superfamily including peptidoglycan degradation, amide and ester bond hydrolysis, and acyl transfer on a variety of substrates [107]. These ubiquitous enzymes can be sub-classified into 4 groups. Representatives of only one of them, LRAT-like proteins, are found in vertebrates [107]. Nine genes that belong to a LRAT-like class are present in the human genome: five HRAS-like tumor suppressors, named after their putative role in decreasing growth of certain tumors, two have yet to be characterized, FAM84A and FAM84B enzymes, and LRAT. The existence of several paralogs of HRASLS and FAM84 enzymes in vertebrate genomes may suggest more diverse functions of these enzymes. Interestingly, genomic searches of invertebrate species revealed numerous putative members of LRAT-like family [106]. However, they retrieved HRASLS enzymes rather than LRAT as best hit in reciprocal BLAST searches. Thus, highly specialized LRAT belongs to a widespread, multigene family of enzymes with several related genes per genome. It is very likely that LRAT emerged as a result of gene duplications from a HRASLS-like ancestor followed by subsequent functional divergence, traditionally considered to be a major evolutionary source of new protein functions in eukaryotes.

### 4.2. Biochemical Properties and Enzymatic Activities of N1pC/P60 Proteins 

Paradoxically, the functional diversity of N1pC/P60 proteins originates from a common structural motif and mechanism for catalysis [107]. As a consequence of the structural relationship to thiol protease, N1pC/P60 enzymes are structurally related to papain and consist of an α-helix that is packed against a four-strand antiparallel β-sheet. The active site is defined by a conserved Cys or Ser residue accompanied by a histidine side chain and an additional polar amino acid [108,109]. Special positions of these residues are preserved with respect to secondary structural elements. Although there are significant topological differences derived from a circular permutation within the catalytic domain of a LRAT-like subfamily, the structural organization implies a common catalytic strategy where the deprotonated sulfhydryl group of a cysteine residue serves as a nucleophile attacking the carbonyl carbon of a substrate [40,108]. Subsequent formation and collapse of short living tetrahedral intermediate result in transient acylation of the enzyme and liberation of the cleavage product. In the second stage of the enzymatic reaction, the active site is regenerated by transferring the acyl moiety onto a water molecule (hydrolysis) or an alternative acyl acceptor (acyl transfer). Thus, the same basic catalytic mechanism allows accommodating numerous enzymatic activities [95]. Consequently, alternative to peptide cleavage activities evolved in LRAT-like protein family. 

Similarly to LRAT, vertebrate HRASLS proteins (with exception of HRASLS5) are bitopic integral membrane enzymes with a single transmembrane helix at the *C*-terminus [110]. Functional analysis of their enzymatic activity revealed that they utilize phospholipids, predominantly phosphatidylcholine as the substrate catalyzing cleavage of the ester bond at the sn-1 or sn-2 position [108]. In the presence of a proper substrate, this esterase activity can be accompanied by a transfer of the acyl moiety onto a variety of enzyme-specific substrates including lyso-phospolipids, phosphatidylethanolamine or short chain alcohols [108,111,112,113]. In contrast, LRAT’s enzymatic activity is highly specific and refined, despite high similarity of the catalytic domain. Esterase activity of the enzyme is not detectable. Instead, LRAT transfers the acyl chain exclusively from PC onto vitamin A or closely related retinoid-like substrates [40,43]. Moreover, LRAT’s activity is restricted towards the sn-1 fatty acid position in the phospholipids substrate [80]. 

In conclusion, highly related lipid membrane associated LRAT-like enzymes display diverse substrate specificity that arise neither from alterations of the catalytic mechanism nor from lipid substrate accessibility. This distinguishing characteristic made LRAT-like proteins an attractive model for structure/function relation studies that shed new light on the molecular evolution that enables esterification of vitamin A. 

### 4.3. Structural Insight into Functional Adaptation of LRAT

**Figure 4:**
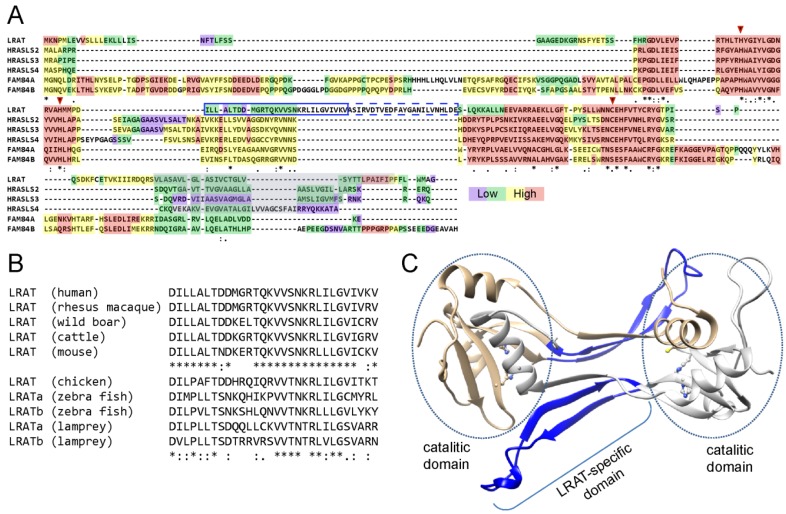
Sequence alignment of representatives of N1pC/P60 proteins family. (**A**) A protein sequence alignment of selected LRAT-like proteins encoded in the human genome. The color scheme (from red to purple) reflects the level of consistence between primary protein sequences. As indicated in the alignment, the catalytic domains of LRAT-like proteins are highly conserved, whereas *N*- and *C*-terminus vary in length and sequence. Conserved His and catalytic Cys/Ser residues are marked with red triangles. Hydrophobic *C*-terminal membrane-anchoring sequences in lecithin:retinol acyltransferase (LRAT) and HRAS-like tumor suppressors (HRASLS2, 3, and 4) are indicated with a gray background. LRAT-specific sequence is marked with a blue rectangle. The solid line corresponds to the sequence stretch that forms an extended linker between β-strand 3 and 4 of the catalytic domain and is indispensable for efficient acyl transfer onto vitamin A. T-Coffee method was used for sequence comparison [80]; (**B**) Conservation of the LRAT-specific domain among a variety of vertebrate species; (**C**) The dimeric structural model of human LRAT. Location of LRAT-specific domain within the structure is marked in blue. The side chains of residues involved in the catalysis are shown as ball and sticks.

The sequence alignment between LRAT and HRASLS proteins clearly indicates existence of a conserved catalytic core of the enzymes followed by a *C*-terminal hydrophobic membrane interacting domain (Figure 4A). Remarkably, all LRAT sequences contain a unique 30 amino acids long insertion right in the middle of the catalytic domain, which is not found in HRASLS proteins (Figure 4B). Although these sequence differences were promptly recognized, proper interpretation of their function was delayed by a lack of adequate structural information about the 3-dimensional architecture of the catalytic domain. The breakthrough happened during determination of HRASLS2 and HRASLS3 structures, which allowed for reevaluating the sequence alignment in the context of the proteins’ architecture and their topology at the lipid membrane. Homology modeling indicated that LRAT’s characteristic sequence forms an extended linker between β-strands 3 and 4 of the catalytic domain (Figure 4C). The amphiphilic character of this region suggests that this part of the enzyme is involved in interaction with the lipid bilayer. The functional significance of this region was demonstrated by constructing recombinant chimeric enzymes in which the 30-aa LRAT-specific sequence was added to the catalytic domain of HRASLS2, 3 or 4 [80]. In contrast to the native HRASLS proteins, all of the tested chimeric HRASLS/LRAT enzymes revealed the ability to transfer the acyl moiety onto vitamin A forming REs. This gain-of-function approach demonstrated that it was possible to change substrate specificity of HRASLS enzymes by replacing a well-defined portion of these enzymes with the LRAT-specific sequence.

The determination of the crystal structure of HRASLS3/LRAT chimeric protein provided a mechanistic explanation for the remarkable change in substrate specificity [80]. The LRAT domain folds into an extended β-hairpin motif that imposes profound structural and functional consequences for the enzyme. Unlike native HRASLS, the chimeric protein forms a constitutive homodimer [80,108]. The major dimerization interface is reinforced by hydrophobic interactions between the side chains of two LRAT domains from the neighboring protomers. The LRAT domain also provides a platform for tertiary conformational rearrangements involving 3-dimentional domain swapping. In consequence, the *C*-terminal α-helix containing a catalytic Cys residue contributes to the active site of adjacent protomers. However, how are these structural changes translated into functioning of the enzyme? The answer to this question can be inferred by a comparison between HRASLS3 and chimeric enzyme structures. Because of the reduced size of the loop between β-strands 3 and 4 and its flexible conformations as shown by NMR data [114], the catalytic Cys residue in HRASLS3 is fully accessible to the solvent. In contrast, the active site environment in the chimera is altered by a hydrophobic pocket formed by LRAT domains. The ultimate consequence of the active site shielding is a limited access of water molecules severely reducing the hydrolysis of the thioester catalytic intermediate. This stabilization of thioester is a necessary prerequisite for efficient acyl transfer onto alternative to water or surrounding phospholipids molecules; a property that distinguishes LRAT from related HRASLS enzymes.

Thus, comparative biochemical analyses of the LRAT-like protein family offered valuable insight into the molecular mechanisms, by which new enzymatic activities can emerge through naturally occurring modifications of preexisting structural motifs. The most profound adaptation of LRAT-like proteins to vitamin A metabolism happened some 500 million years ago in vertebrate linage by insertion of a 30-aa β-hairpin into the canonical catalytic domain of a common LRAT ancestor.

## 5. Mutations in *LRAT* Gene and Their Clinical Manifestations

The physiological significance of vitamin A esterification places LRAT at the crossroads of retinoid uptake and metabolism. Consequently, mutations in *LRAT* gene that lead to a reduced function or even to a complete inactivation of the enzyme are associated with human diseases. Functional analysis of mouse mutants and the phenotype of afflicted patients with inherited *LRAT* mutations provide important insights about vitamin A homeostasis in the absence of functional LRAT. 

### 5.1. Role of LRAT in the General Retinoid Homeostasis

Bearing in mind the significance of retinoids in regulation of the immune system, reproduction or development, and high expression of LRAT in many vital tissues, the lack of LRAT activity was expected to be lethal. Remarkably, *Lrat^−/−^* mice are born alive and develop normally [25,60]. However, with time, their physical appearance becomes distinguishable from WT mice. They display signs of craniofacial abnormality characterized by shortened and wider faces. Although this phenomenon has not been properly scrutinized, observed morphological aberrations might be an effect of imbalanced vitamin A homeostasis on cranial bones’ growth. In fact, the dominant phenotype of *Lrat^−/−^* is the near absence of fatty acid REs in the liver, lung, kidney, and eye [25]. Interestingly, when kept on a vitamin A rich diet, these mice maintain serum vitamin A levels similar to WT animals. However, serum retinol concentration is prone to dramatic changes depending on dietary intake [59,115]. Ten-fold higher food vitamin A supplementation caused a rapid increase in serum retinol concentration [115]. Notably, these high levels of retinol persisted at least 2 weeks after switching back to the regular diet. Excess vitamin A resulted in abnormal distribution of retinol between blood and peripheral tissues causing elevated retinoic acid levels and subsequent activation of cytochrome P450 family 26 subfamily A member 1 (CYP26A1)-mediated retinoid catabolism [59,60,115]. These effects could not be effectively mitigated by the alternative pathways of retinoids secretion such as increased gastrointestinal excretion, the re-direction of retinol to adipose tissue and its esterification via an acyl-CoA-dependent mechanism. In contrast, after maintaining a retinoid-deficient diet for 6 weeks, *Lrat^−/−^* mice had significantly lower serum retinol levels than WT mice and undetected levels of retinol in a number of tissues [59].

The role of LRAT in preventing retinoic acid toxicity was elegantly demonstrated in a zebrafish model [116]. Acyltransferase activity of Lratb in wild-type zebra fish embryos is sufficient to maintain retinoic acid homeostasis in the presence of excess exogenous retinol. Concurrently, a knock down of Lratb resulted in a decrease of REs accompanied by the elevation of retinoic acid concentration to the level incompatible with normal embryonic development. Thus, competition for a substrate between REs and retinoic acid synthesizing pathways dynamically influences retinoic acid levels during early embryonic development in this oviparous vertebrate. 

The iconic example of the role of LRAT in maintaining vitamin A homeostasis is retinoic acid-dependent regulation of expression of this enzyme in liver and lungs. The initial studies by Dr. Ross and colleagues indicated that LRAT activity was much lower in the liver of vitamin A deficient rats as compared to the control animals. Interestingly, LRAT activity could be induced by retinoic acid or supplemental vitamin A [117,118]. The same phenomenon applied to other tissues including lungs but not small intestines and it is conserved in mice [119,120]. The elevated LRAT activity was attributed to induction of LRAT expression leading to a higher concentration of the enzyme rather than through activation of the enzyme by an allosteric mechanism or a posttranslational modification [120]. Importantly, increased accumulation of REs in the tissues was accompanied with significant decrease in plasma retinol levels in rats 16 h after retinoic acid administration. These observations led to a model for regulation of vitamin A homeostasis, in which the baseline expression of liver LRAT during retinoid sufficiency serves to divert retinol into storage pools, while diminished LRAT expression in retinoid deficiency would promote secretion and accessibility of retinol to other tissues [121]. Thus, this mechanism may contribute to relatively mild systemic phenotype of *Lrat^−/−^* kept on vitamin A sufficient diet. Altogether, these data strongly indicate that LRAT is not only important for storing retinol as REs in the liver, but is also involved in maintaining a stable retinol concentration in the serum of adult mice. In addition, LRAT-dependent buffering of vitamin A concentrations in mammals seems to be critical for maintaining proper retinoic acid signaling. 

It is important to note that although the general role of LRAT in vitamin A homeostasis is conserved among vertebrates there are remarkable differences among species in how retinoids are distributed among tissues, mobilized or transported in the circulation in response to the shortage of dietary vitamin A. Studies on domesticated carnivores indicated that unlike in human, mouse, and rats the dominant form of retinoid in fasting dogs, cats, and ferrets is not retinol but rather REs [122,123,124]. The differences in the blood retinoid composition can be seen even between closely related species. In orangutans and chimpanzees plasma REs constitute up to 20% of total retinoids, whereas only 5% in humans [125]. Despite limited data and lack of biochemical basis for these discrepancies, it is tempting to speculate that they are related to the feeding behaviors (carnivore vs. omnivore/herbivores) that determine source, relative amount, and bioavailability of vitamin A or its precursors. In addition to the retinoid composition in serum, there are well documented differences in concentration of REs in the peripheral tissues. The best example are lungs, where the amount of REs per gram of tissue is comparable with liver in mice but nearly 100 times lower in humans or even rats [22,60,126]. Thus, these fundamental physiological variances may directly contribute to higher susceptibility for vitamin A deficiency of some species versus others. 

### 5.2. Synthesis of REs Incorporated into Milk

In addition to the essential role in vitamin A homeostasis, LRAT contributes to the retinoid composition of milk secreted from lactating mammary glands [127]. At birth, newborns do not possess sufficient retinoid storage to support normal growth and development and are thus dependent on their mother’s milk for acquiring enough retinoids. REs constitute more than 95% of total retinoids found in the milk of WT mice. The retinoid composition of milk obtained from *Lrat^−/−^* mice revealed reduced amounts of REs that was however compensated by an increased concentration of free retinol [60]. Additionally, the remaining pool of vitamin A esters contained medium fatty acid chains. This fact strongly suggests the contribution of the alternative to LRAT, acyl-CoA-dependent enzymes in their production. Although LRAT is the predominant enzyme responsible for the formation of milk REs, mammary tissue show surprising adaptations to maintain proper levels of milk retinoids, even in conditions that adversely affect availability of vitamin A by other tissue. 

### 5.3. Eye Phenotype in LRAT-Deficiencies

In addition to impaired retinoid accumulation, perhaps the most dramatic phenotype of *Lrat^−/−^* mice is manifested in the eye. Consistently with the role of LRAT in the retinoid cycle, these genetically modified animals are incapable of producing the visual chromophore, 11-*cis*-retinal [25]. The lack of the functional visual pigment causes fast-progressing degeneration of the retina that morphologically is evident by initial shortening of the rod outer segments followed by disorganization of photoreceptor cells’ synaptic terminals. Electrophysiological photoresponses in scotopic and photopic conditions indicate that both rod and cone functions of *Lrat^−/−^* mice are dramatically affected [25]. Interestingly, LRAT deficiency seems to cause a more pronounced phenotype than *Rpe65^−/−^* mice [128]. This fact might be attributed to the absence of retinoid retention in RPE cells where only traces of vitamin A and virtually no REs can be detected. The ocular phenotype characteristic for *Lrat^−/−^* mice is to a certain degree recapitulated in patients affected by mutations in *LRAT* gene. Thus, *Lrat^−/−^* mice served as an animal model for early onset severe retinal dystrophy in studies aimed at the development of visual chromophore replacement or gene therapies for this blinding disease. 

### 5.4. Clinical Manifestation of Mutations in LRAT Gene

Severe visual impairment is the primary cause for seeking medical attention for patients affected by LRAT deficiency. The onset of visual disorders is usually reported between two months and three years of age, in otherwise healthy patients. The universal features of the impairment are the inability to see in dim light conditions (nyctalopia), progressive deterioration of color vision, and loss of their visual field. Macroscopic appearances of the retina in the early stage of the disease typically do not reveal anatomic abnormalities [129]. However, widespread bilateral RPE atrophy arteriolar attenuation, sub-retinal pigmentation, foveal and optic nerve atrophy become evident in the second decade of life. The electroretinogram (ERG) recordings from the retina reveal a highly attenuated residual response characterized by increased threshold and reduced amplitude. In advance stages of retinal degeneration, rod-mediated full-field ERGs are usually undetectable, whereas ERG recordings from cones show only minor residual responses [130,131,132]. 

Interestingly, there are no reports about obvious signs characteristic for systemic vitamin A such as Bitot’s spots, corneal changes or growth retardation deficiency in LRAT-deficient patients with advanced retinal degeneration. Although they were not screened for plasma concentrations of circulating retinol, retinyl esters, retinoic acid or β-carotene and thus nothing is known about their vitamin A status, it is conceivable that adequate dietary intake of pro-vitamin A carotenoids could supply a sufficient amount of retinoids to sustain function of most tissues except for the retina [133,134].

For the most part, the above description of retinal pathologies is not unique for LRAT deficiencies. They can be observed in retinopathies caused by mutations in other genes related to the development or function of the retina. At least 14 genes are associated with early-onset severe retinal dystrophy causing difficulties in proper phenotypic classification without in depth genetic tests [129]. Thus, this variety of blinding retinal diseases is often classified as Leber Congenital Amaurosis (LCA). Although molecular mechanisms of LCA may not be fully understood for mutations in many of the associated genes, in the case of LRAT, the inability to retain retinoids in the RPE and resulting lack of the visual chromophore appears sufficient to precipitate disease progression. This well-defined pathogenesis makes LRAT defects amenable to retinoid replacement therapy [135]. As evident by studies on LCA mouse model, oral treatment with 9-*cis*-retinal that can substitute natural 11-*cis* isomer, dramatically improved retina function and preserved its cellular architecture [136]. Similar results were obtained using chemically inert and more tolerable 9-*cis*-retinyl acetate or 9-*cis*-retinyl succinate that serve as pro-drug forms of 9-*cis*-retinal [137]. In the initial clinical trial (phase 1b) oral administration of 40 mg/m^2^ of 9-*cis*-retinyl acetate (QLT091001) for seven consecutive days resulted in expansion of functional retinal area in 71% of enrolled LCA patients, 43% displayed improved visual acuity [138,139]. Importantly, these functional changes were associated with healthier retinal morphology measured as thinness of photoreceptor cell outer segments. Thus, this mechanism-based pharmacological intervention that bypasses metabolic block in vitamin A metabolic pathway has the potential to preserve and restore vision in otherwise incurable genetic retinal degeneration.

### 5.5. Impact of Retinal Diseases-Associated LRAT Mutations on Structure and Activity of the Enzyme

One of the characteristics of eye diseases caused by mutations in LRAT is heterogeneity of the clinical conditions. These phenotypic differences most likely originate from effects of the mutation on the enzymatic activity of LRAT. Currently, there are 13 identified mutations in *LRAT* gene that cause LCA or milder retinitis pigmentosa in humans (Table 1), six of which result in reading frame shifts, incorporation of unrelated to wild-type sequence, and pre-mature termination of translation, whereas the remaining seven represent single amino acid substitutions. Although effects of these mutations on LRAT’s activity have not been studied systematically, it is easy to envision that an incomplete polypeptide chain has more of an obvious impact on protein dysfunction. For example, three of the frame shift mutations 12delC, 217_218delAT, 397_398delAA, and 427_428delCG produce a truncated enzyme that lacks the conserved NCEHFV domain with catalytic Cys161 residue. In fact, patients affected by these inactivating deletions suffer from early-onset and more severe retinal dystrophy [132,139,140,141]. Interestingly, mutations 519delG and 613_614delCT do not interfere with the catalytic domain but eliminates the *C*-terminal transmembrane helix [142,143]. Biochemical data clearly indicate that LRAT without the transmembrane domain remains catalytically active [40,144]. However, when expressed in COS-7 cells, the truncated enzyme localized to small cytoplasmic structures that were distinct from the endoplasmic reticulum and dispersed throughout the cell body [110]. Thus, *C*-terminal domain is necessary for endoplasmic reticulum targeting and anchoring of LRAT. Intracellular localization of LRAT is also detrimental for access to lipid substrates. 

**Figure 5:**
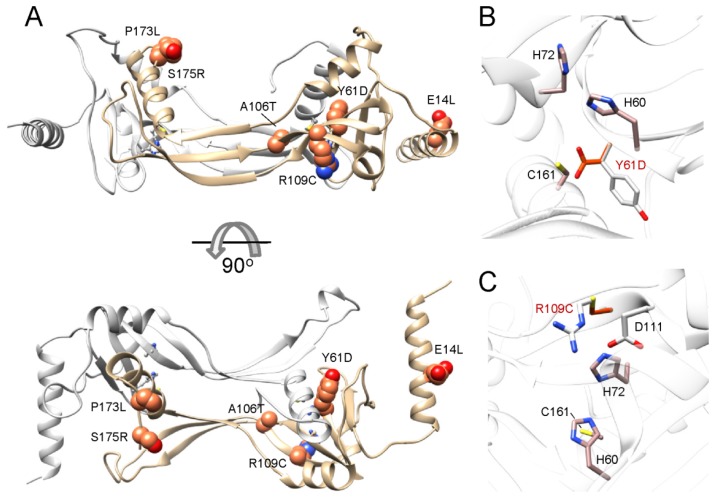
Location of pathogenic mutations within the LRAT structure. (**A**) Substituted residues associated with retinal degenerative diseases (shown as spheres and colored in orange) are predominantly clustered within the catalytic domain with exception of L14E localized in the *N*-terminal helix; Panels (**B**,**C**) represent close-up views of residues Y61 and R109, respectively. Their proximity to the active site may indicate mutations affecting these amino acids might disrupt the active site geometry and thus disable the enzyme. Residues that substitute native amino acids are shown in red. Residues involved in the catalysis are colored in light brown.

The consequences of protein truncation can be inferred directly from the primary sequence. Elucidation of functional consequences of single amino acid substitutions requires a 3D structural framework. Crystal structure of HRASLA3/LRAT allows for understanding mechanisms by which mutations in LRAT might affect its activity (Figure 5A). This is particularly important in the absence of biochemical data validating enzymatic activity of mutated proteins. In fact, only the S175R mutation was experimentally confirmed to lead to inactivation of LRAT with an expression level equivalent to WT. From a structural point of view, S175 is located outside the catalytic domain in a linker connecting α-helix 4 containing catalytic C161 with the transmembrane domain. Replacement of small, polar serine for a much larger positively charged arginine residue could potentially impose destabilizing effects on α-helix 4 or β-strand 6 at the dimer interface leading to alteration of the active site architecture and loss of the enzymatic activity. Interestingly, nearby P173L substitution is also pathological causing retinitis pigmentosa. Proline is well known as a helix breaker and is commonly found in turns, as it is in LRAT. Hence, its replacement by leucine, which has stabilizing effects on α-helix, is expected to affect the length of α-helix 4 and thus size of the linker connecting the transmembrane domain. The last mutation found in this region is substitution R190H [145]. It occurs in a cluster of charged residues (RDQR) that forms the *N*-terminal cup flanking the transmembrane helix. 

Another group of mutations (Y61D, A106T, and R109C) is localized in close vicinity to the active site: Tyrosine at the position 61 follows directly the catalytic H60 and spatially neighbors C161 (Figure 5B). Mutations in this site could disrupt packing of α-helix 4 against the β-sheet, which in turn, may cause displacement of C161 side chain with respect to the orientation of H60, preventing deprotonation of SH group. A similar mechanism applies to R109C [145] (Figure 5C). The active site architecture is restrained by hydrogen bond interactions between H60, H71, and R109. Dramatic substitution of R109 by a cysteine residue destroys this network of hydrogen bonds leading to re-orientation of the histidine side chains and consequently inefficient catalysis. It is important to note that a mechanism, in which some of the disease causing mutations affects LRAT activity, cannot be inferred directly from the available structural information. For example, substitution of A106T represents a minor addition of a hydroxyl group that changes polarity of the side chain. Although this mutation occurs in the vicinity of the hydrophobic pocket holding an acyl moiety, it is unclear why it imposes such a dramatic effect on the enzyme. Even more mysterious is the E14L mutation. The *N*-terminal membrane-interacting α-helix exhibits amphiphilic properties with discrete polar and hydrophobic sides. Moreover, LRAT’s *N*-terminus is not required for proper protein localization within the cell or the activity; nevertheless patients affected by this mutation suffer from a severe form of LCA associated with atrophy of RPE cells [130]. The plausible mechanisms involve misfolding and degradation of the mutated protein, however this severe phenotype may also indicate yet to be discovered functions of *N*-terminal helix. 

It is important to realize that the described above molecular mechanisms of LRAT inactivation might be amplified by homo-dimerization, domain swapping, and membrane localization of the enzyme. Nevertheless, further evaluation of the mechanisms that precipitate retinal pathologies will require additional functional studies in combination with determination of a genuine LRAT structure.

## 6. Conclusions and Future Directions

There is no doubt that over the last two decades, we have witnessed tremendous improvement in our understanding of retinoid metabolism. This progress has been driven by identification of novel proteins and the generation of numerous mouse models deficient in genes involved in vitamin A metabolism. In parallel with these efforts, structural biology provided valuable knowledge regarding molecular organization and a mode of action for retinoid-binding proteins [79], RPE65 [91,92], and LRAT [80]. Despite this indisputable progress, each new discovery raises additional questions reminding us how much more we need to learn about retinoid metabolism throughout the body. The following is just a list of few questions that in our opinion represent the current challenges in the field.

(a) Concentration of retinol in the blood correlates with vitamin A status of the organism only in cases of depletion or over saturation of the liver storage. Between these two extremes vitamin A level is homeostatic. What is the signaling pathway in which vitamin A concentration in the blood regulates mobilization of retinoid pool in the liver? Is this process controlled on the transcription level or maybe involves allosteric regulation of enzymes involved in retinyl esters hydrolysis/formation?
(b) A related unsolved problem is how retinol is trafficked from hepatocytes to lipid storage droplets in HSCs. In addition, unclear is the enzymatic machinery responsible for mobilization of retinyl esters storage and how it is regulated.
(c) Uptake of vitamin A to peripheral tissue is also a controlled process. For example, excess of all-*trans*-retinol does not cause disproportionate accumulation of retinyl esters in the RPE. However, in vitamin A deficient states the ocular retinoid uptake is favored over other tissues [71,77]. Although the pivotal role of STRA6 and LRAT in vitamin A uptake is unquestionable, it seems that transcriptional regulation of STRA6 and LRAT expression by retinoic acid is not the only mechanism that governs “buffering” of vitamin A within the eye or other tissue. The components of this signaling pathway remain to be uncovered.
(d) One of the contemplated biochemical questions in retinoid metabolism is the cone-specific visual chromophore regeneration pathway. Alterations in retinoid composition between nocturnal and diurnal animals as well as electrophysiological differences in rod and cones responses suggest the existence of an alternative to the RPE65 source of 11-*cis*-retinal that supports cone function. Although putative enzymes involved in this pathway have been identified [146,147], their physiological functions remain to be verified in in vivo models.
(e) Despite wealth of biochemical and structural data, it is still unknown what triggers controlled release of retinol from retinol-binding proteins. Vitamin A needs to be transported from the plasma membrane or its storage sites to specific compartments within the cell. It is clear that this cannot be a stochastic event but rather a controlled process facilitated by specific interactions of retinol-binding proteins with phospholipids or protein binding partners, which are still unknown.
(f) From the clinical point of view, the successful fight against vitamin A deficiency depends on reliable methods of assessing retinoid status of individuals. Unfortunately, serum retinol is not useful for this purpose. Thus, development of dependable and inexpensive diagnostic tests based on biomarkers other than serum retinol would have tremendous impact on assessing the prevalence, prevention, and treatment of vitamin A deficiencies.

## Figures and Tables

**Table 1:** Mutations in *LRAT* gene associated with retinal degeneration.

Nucleotide Change	AA Mutation	Exon	Reference
12 ^1^ del ^2^ C	P4fs ^3^ (53)	2	[141]
40_41delGAins ^4^ TT	E14L	2	[130]
T181A	Y61D	2	[130]
217_218delAT	M73fs(47) ^4^	2	[132]
G316A	A106T	2	[130]
C325T	R109C	3	[145]
397_398delAA	K134fs(11)	3	[131,140]
427_428delCG	R143fs(2)	3	[139]
C518T	P173L	3	[132]
519delG	I174fs(11)	3	[142]
A523T	S175R	3	[131,140]
G569A	R190H	3	[145]
613_614delCT	S205Yfs(27)	3	[143]

^1^ nucleotide numbers were normalized and correspond to the cDNA sequence; ^2^ del: deletion; ^3^ fs: frame shift, number in brackets represent missense amino acids before stop codon; ^4^ ins: insertion.

## Abbreviations

The following abbreviations are used in this manuscript:

BCO1	β,β-carotene-15,15-dioxygenase
CRALBP	cellular retinaldehyde-binding protein
CRBP1	cellular retinol-binding protein 1
CRBP2	cellular retinol-binding protein 2
CYP26A1	cytochrome P450 family 26 subfamily A member 1
DGAT1	diacylglycerol *O*-acyltransferase 1
ER	endoplasmic reticulum
ISX	intestinal specific homeodomain transcriptional factor
HRASLS2	HRAS-like tumor suppressor 2
HRASLS3	HRAS-like tumor suppressor 3
HSCs	hepatic stellate cells
LCA	leber congenital amaurosis
LRAT	lecithin:retinol acyltransferase
RARs	retinoic acid receptors
RBP	serum retinol-binding protein
RDH	retinol dehydrogenase
REs	retinyl esters
RPE	retinal pigmented epithelium
RPE65	retinal pigment epithelium-specific 65 kDa protein or retinoid isomerase
RXRs	retinoid X receptors
SCARB1	scavenger receptor class B, type I
STRA6	stimulated by retinoic acid 6
TTR	transthyretin
WT	wild-type
